# Role of the Placental Vitamin D Receptor in Modulating Feto-Placental Growth in Fetal Growth Restriction and Preeclampsia-Affected Pregnancies

**DOI:** 10.3389/fphys.2016.00043

**Published:** 2016-02-18

**Authors:** Padma Murthi, Hannah E. J. Yong, Thy P. H. Ngyuen, Stacey Ellery, Harmeet Singh, Rahana Rahman, Hayley Dickinson, David W. Walker, Miranda Davies-Tuck, Euan M. Wallace, Peter R. Ebeling

**Affiliations:** ^1^Department of Medicine, School of Clinical Sciences, Monash UniversityMelbourne, VIC, Australia; ^2^Department of Obstetrics and Gynaecology, School of Clinical Sciences, Monash UniversityMelbourne, VIC, Australia; ^3^Department of Obstetrics and Gynaecology, The University of MelbourneMelbourne, VIC, Australia; ^4^Department of Maternal-Fetal Medicine Pregnancy Research Centre, The Royal Women's HospitalMelbourne, VIC, Australia; ^5^The Ritchie Centre, Hudson Institute of Medical ResearchMelbourne, VIC, Australia

**Keywords:** VDR, fetal growth restriction, trophoblast, placental transport

## Abstract

Fetal growth restriction (FGR) is a common pregnancy complication that affects up to 5% of pregnancies worldwide. Recent studies demonstrate that Vitamin D deficiency is implicated in reduced fetal growth, which may be rescued by supplementation of Vitamin D. Despite this, the pathway(s) by which Vitamin D modulate fetal growth remains to be investigated. Our own studies demonstrate that the Vitamin D receptor (VDR) is significantly decreased in placentae from human pregnancies complicated by FGR and contributes to abnormal placental trophoblast apoptosis and differentiation and regulation of cell-cycle genes *in vitro*. Thus, Vitamin D signaling is important for normal placental function and fetal growth. This review discusses the association of Vitamin D with fetal growth, the function of Vitamin D and its receptor in pregnancy, as well as the functional significance of a placental source of Vitamin D in FGR. Additionally, we propose that for Vitamin D to be clinically effective to prevent and manage FGR, the molecular mechanisms of Vitamin D and its receptor in modulating fetal growth requires further investigation.

## Introduction

Fetal growth restriction (FGR) is defined as the failure of a fetus to reach its full growth potential for its gestational age, and complicates up to 5% of all pregnancies (McIntire et al., 1999; Scifres and Nelson, 2009). A low birth weight, accompanied by additional pathologies such as asymmetric growth and reduced amniotic fluid index, allows clinicians to distinguish FGR cases from infants who are born small for gestational age, but are otherwise healthy. FGR is associated with increased risks of perinatal complications such as prematurity (Gardosi et al., 1998) and stillbirth (Kramer et al., 1990; Cnattingius et al., 1998; Gardosi et al., 1998; Froen et al., 2004), in addition to an increased risk of mortality (Kramer et al., 1990; McIntire et al., 1999). Long-term adverse outcomes include impaired neuropsychological development (Taylor and Howie, 1989; Frisk et al., 2002), reduced intelligence quotients (Goldenberg et al., 1996; Matte et al., 2001) and poor metabolic health with greater risk of chronic adulthood diseases including hypertension, ischemic heart disease, and diabetes (Barker, 1990; Godfrey and Barker, 2001). Thus, FGR has potential lifelong consequences for an infant (Ponsonby et al., 2010).

While there are several factors attributed to the causes of FGR, including fetal chromosomal abnormalities, placental infarcts, maternal tobacco smoking, and poor nutrition; for the majority of cases, ~70%, there is no known cause (Sankaran and Kyle, 2009). Idiopathic FGR is often associated with uteroplacental vascular insufficiency, whereby there is abnormal placental development and function, which leads to impaired vascular flow to the placenta (Robinson et al., 2000). Sampling of the umbilical cord blood from growth restricted infants shows chronic and inadequate transplacental oxygenation from mother to child (Ghidini, 1996). Clinical features of poor placental perfusion are abnormal umbilical artery Doppler velocimetry such as decreased, absent or even reverse diastolic flow (Salafia et al., 1997), oligohydramnios (Harman, 2008), and asymmetric fetal growth (Vik et al., 1997). The molecular mechanisms underlying such uteroplacental insufficiency remain largely unknown.

## Vitamin D metabolism, transport, and bioavailability

Vitamin D exists in two forms; Vitamin D_3_ and Vitamin D_2_, both of which have very similar structures. Vitamin D_3_ contributes to 95% of the Vitamin D levels in the human circulation (Holick, 2003). The major source of Vitamin D in humans is the cutaneous synthesis of pre-Vitamin D_3_, which is derived from 7-dehydrocholesterol through exposure to ultraviolet radiation (Holick, 2007). Vitamin D_3_ varies with the level of sunlight exposure and is extremely susceptible to seasonal changes (Holick, 2007). Vitamin D stores can be built up during summer months and utilized as serum levels drop during the winter (Holick, 1994; Heaney, 2005; Heaney et al., 2005). In contrast, Vitamin D_2_, which is a third as potent as Vitamin D_3_, is found in some plants, dietary supplements, and multi-vitamins (Norman, 2012). Major dietary sources of Vitamin D include oily fish, fortified margarines and some breakfast cereals, while smaller amounts are present in red meat and egg yolk (Atkinson, 2011), although these contribute only small amounts compared to endogenous synthesis.

Vitamin D (either D_2_ or D_3_) made in the skin or ingested in the diet can be stored in and then released from fat cells. Both forms of Vitamin D are biologically inactive and must undergo two sequential hydroxylations by mitochondrial enzymes known as 25-hydroxylase (CYP2R1) and 1-α-hydroxylase (CYP27B1) to become calcidiol [25(OH)D] and calcitriol [1,25(OH)_2_D] respectively (Holick, 2007). Vitamin D in the circulation binds to the Vitamin D–binding protein, which then transports it to the liver, where it is converted by CYP24A1, a 24-hydroxylase mitochondrial cytochrome p450 enzyme, to a pre-Vitamin D, 25(OH)D. This form of Vitamin D is biologically inactive and must be converted in the kidneys by 1α- hydroxylase (1-OHase) to the biologically active form, 1,25-dihydroxyVitamin D[1,25(OH)_2_D]. Serum phosphorus, calcium, fibroblast growth factor 23 (FGF-23), and other factors alter the renal production of 1,25(OH)_2_D. 1,25(OH)_2_D increases the expression of 25-hydroxyvitamin D by Vitamin D-24- hydroxylase (24-OHase) to catabolize 1,25(OH)_2_D to the water-soluble, biologically inactive calcitroic acid, which is then excreted in the bile. The CYP24A1 hydroxylase enzyme also converts substrates into inactive end products, including 1,24,25-trihydroxyvitamin D and 24,25-dihydroxyvitamin D (Omdahl et al., 2002). As a protective mechanism to prevent Vitamin D intoxication (due to excessive exposure to sunlight), pre-Vitamin D_3_ and Vitamin D_3_ can be converted to inactive products by solar ultraviolet radiation (Holick, 2007).

## Vitamin D receptor (VDR)

Vitamin D has limited effects on most target cells and its biological activity often correlates with free Vitamin D concentration, where the unbound form of 1,25(OH)_2_D diffuses across the plasma membrane and binds to its high affinity nuclear receptor, VDR. VDR can mediate both non-transcriptional and transcriptional effects. In the transcriptional pathway, 1,25(OH)_2_D bound VDR heterodimerises with a partner receptor, retinoid X receptor (RXR). This VDR-RXR complex regulates the transcription of Vitamin D target genes by binding with high affinity Vitamin D response element (VDRE) located in the promoter region, resulting in a cascade of cellular events (Avila et al., 2007). On the other hand, the non-transcriptional pathway involves calcitriol-VDR complex binding with caveolae stimulating numerous signaling cascades, such as protein kinase C and mitogen-activated protein kinase (Shin et al., 2010). These signaling cascades play a pivotal role in regulating cellular functions such as proliferation, differentiation, invasion, and apoptosis hence highlighting the modulatory effects of Vitamin D. VDR allelic variants and altered VDR expressions are associated with a number of health concerns including prostate cancer (Ingles et al., 1997), breast cancer (Whitfield et al., 2001), and sarcoidosis (Niimi et al., 1999), but the role of VDR and Vitamin D signaling in pregnancy is poorly understood.

## Vitamin D homeostasis in pregnancy

Vitamin D is a pleiotropic secosteroid hormone, which is pivotal for the maintenance of calcium homeostasis, bone mineralization, immune regulation, cellular proliferation, and prevention of disorders (Norman, 2012). Vitamin D status is reflected by the circulating 25(OH)D concentrations, for which the normal range is defined to be between ~ 32ng/mL and ~ 80ng/mL, with values below ~32 ng/mL is defined as Vitamin D deficiency (Holick, 2007).

Emerging evidence in observational studies indicate that a low circulating 25(OH)D concentration is associated with increased risks of infertility, preeclampsia and gestational diabetes mellitus. Vitamin D deficiency has also been linked to early on-set preeclampsia associated with FGR (Robinson et al., 2010). A number of large observational studies report a significant link between low maternal serum Vitamin D and an increased risk of preeclampsia (Bodnar et al., 2007; Fischer et al., 2007; Haugen et al., 2009; Baker et al., 2010; Robinson et al., 2010; Yu et al., 2013). Bodnar et al. (2007) conducted a nested case control study following pregnant women from less than 16 weeks gestation to delivery and correlated maternal Vitamin D status with the risk of developing preeclampsia, finding that serum 25(OH)D levels early in pregnancy were significantly lower in women who went on to develop preeclampsia than in controls (Bodnar et al., 2007). Similarly, Haugen et al. (2009) collected data from 23,423 pregnant women in Norway and calculated the Vitamin D intake throughout pregnancy via questionnaires, finding that women who took Vitamin D supplementation were 27% less likely to develop preeclampsia than those who took no supplementation (Haugen et al., 2009). The results of these studies were supported by Baker et al. (2010) who, after conducting a nested case control study of 3992 pregnant women, found that maternal Vitamin D deficiency was found to be associated with an increased risk (OR 5.41) of severe preeclampsia (Baker et al., 2010).

In contrast to these studies, Yu et al. (2013) conducted a case-control study of 150 singleton pregnancies who developed preeclampsia and 100 unaffected controls and investigated if Vitamin D levels at 11–13 weeks gestation were altered in those who developed preeclampsia (Yu et al., 2013). The results of this study indicated that first trimester serum Vitamin D levels are not different for pregnancies that subsequently developed late on-set preeclampsia. As such, it was proposed that identifying specific cases of preeclampsia based on severity and gestation may lead to a better understanding of the pathogenesis. This is supported by a number of studies which have found an association between maternal Vitamin D and early onset and severe preeclampsia, but no association between maternal Vitamin D and preeclampsia risk overall was observed. Robinson et al. reported significantly decreased maternal Vitamin D in early onset severe preeclampsia associated with FGR compared to healthy controls (Robinson et al., 2010). Similarly Bodnar et al. found an association between maternal Vitamin D and cases of severe preeclampsia (Bodnar et al., 2014). Low Vitamin D levels have been associated with vascular endothelial cell inflammatory response, increased cytokine mediated suppression of vascular endothelial function and reduced endothelial Vitamin D receptor (VDR) expression (Tarcin et al., 2009; Wei et al., 2013).

## The role of the placenta in vitamin D metabolism

While the specific mechanisms of Vitamin D during pregnancy are not fully understood, Vitamin D undeniably plays an important role in implantation, placentation, and maintenance of a healthy pregnancy. The placenta, which lies between mother and fetus, is the primary site of waste removal, nutrient and gas exchange, and together with the decidua has an important role in Vitamin D metabolism during pregnancy. During human pregnancy, the conversion of 25(OH)D to 1,25(OH)_2_D is increased in the placenta as well as the maternal kidney and decidua (Weisman et al., 1979). The maternal decidua makes 1,25(OH)_2_D and 24,25(OH)_2_D while the placenta synthesizes 24,25(OH)_2_D (Weisman et al., 1976). The inactive 24,25(OH)_2_D synthesized by the placenta accumulates in fetal bone and significantly contributes to skeletal ossification (Weisman et al., 1976). DNA methylation, histone modifications and non-coding RNAs can also affect gene expression in the placenta. Thus, altered genomic imprinting of VDR has effects on both fetal and placental development (Nelissen et al., 2011). However, very little is known about the impact of external (environmental) influences on the Vitamin D system in pregnancy, or the possible genetic and epigenetic variations that might affect Vitamin D homeostasis at the feto-maternal interface and their effects on overall feto-placental growth and development. In addition, the expression of the gene (*CYP24A1*) regulating the enzyme involved in maintaining homeostatic levels of 1,25(OH)_2_D is decreased by DNA methylation (Novakovic et al., 2009).

The human placenta expresses all of the metabolic components associated with Vitamin D signaling, including VDR, RXR, CYP27B1, CYP24A1, and CYP2R1 (Ma et al., 2012). Vitamin D modulates implantation and placentation via two mechanisms; it increases the expression of the homeobox gene *HOXA10* and increases the immune tolerance toward the embryo. The increase in *HOXA10* expression enhances the endometrium's responsiveness to the embryo and aids implantation (Du et al., 2005). In the human syncytiotrophoblast, Vitamin D and its components act together to enhance the expressions of human chorionic gonadotropin (hCG), human placental lactogen, estrogen and progesterone (Shin et al., 2010).

Expression levels of Vitamin D metabolic components also change significantly during pregnancy (Kumar et al., 1979; Paulson and Deluca, 1986). In the first- and second- trimester of a normal human pregnancy, expression of CYP27B1 and VDR is significantly increased over ten-fold in the placenta and decidua compared with the endometrium (Fischer et al., 2007; Novakovic et al., 2009). In particular, VDR is strongly expressed across gestation in both the cytotrophoblast and syncytiotrophoblast (Pospechova et al., 2009; Ma et al., 2012). Notably, gene polymorphisms of *VDR* and its metabolites are associated with several forms of cancer (including lung, breast, colorectal, and prostate cancer), multiple sclerosis, chronic obstructive pulmonary disease, and type I diabetes (Shin et al., 2010). Some of these polymorphisms alter circulating levels of 25(OH)D and disrupt normal Vitamin D actions (McGrath et al., 2010).

The expression of the metabolic components of Vitamin D, including placental VDR, has been reported by many studies. Zehnder et al. demonstrated that mRNA expression of 1α-hydroxylase was found to be greater in the first and second trimester than in third-trimester placentae, whereas the mRNA expression of *VDR* was consistent and pronounced throughout gestation (Zehnder et al., 2002). VDR is an essential component of the Vitamin D metabolic pathway, whereupon activation regulates and initiates the expression of numerous genes involved in cell proliferation and differentiation (Samuel and Sitrin, 2008). Furthermore, VDR expression in the placenta is finely tuned during pregnancy, indicating its eminent role in the development of the placenta and the fetus (Shahbazi et al., 2011).

## Vitamin D deficiency in fetal development

Birth cohort studies are an invaluable resource for studies of the influence of the fetal environment on health in later life. However, to what extent maternal vitamin D status influences fetal development remains uncertain. In a recent study by Hart et al. (2015) examined for the relationship between maternal vitamin D deficiency at 18 weeks' pregnancy and the long-term health outcomes of offspring who were born in Perth, Western Australia, in 1989–1991 by using a cohort of 901 mother-offspring pairs from the Western Australian Pregnancy Cohort [Raine] Study (Hart et al., 2015). The authors reported that Vitamin D deficiency as defined by the circulating serum concentrations of [25(OH)D] < 50 nmol/L was observed in 36% of the pregnant women included in this study. Maternal Vitamin D deficiency during pregnancy was correlated with the offspring's development at various age groups for impaired lung development, neurocognitive disorders, eating disorders in adolescence, bone mass (Hart et al., 2015). This study concluded that sufficient maternal Vitamin D is a critical factor during fetal development *in utero*, more specifically for the optimal development of multiple organ systems. These observations also suggest that Vitamin D may have a pivotal, multifaceted role in the development of fetal lungs, brain, and bone.

## Placental vitamin D and its contribution to FGR

We have recently demonstrated that placental vitamin D content, as well as placental VDR expression, are decreased in human FGR and contribute to trophoblast dysfunction (Nguyen et al., 2015a). Therefore, decreased placental VDR expression may impair or limit the beneficial actions and effects of maternal/placental Vitamin D in the regulation of feto-placental growth. However, our results did not clarify whether the changes associated with placental VDR expression in FGR pregnancies are due to cause or a consequence of the pathophysiological defects observed in the FGR-affected placentae.

Genetic and epigenetic factors are known regulators of Vitamin D and its metabolic components. Therefore, they may have a significant influence on feto-placental development (Sandovici et al., 2012). Previous studies have reported that the placental Vitamin D metabolic components are tightly regulated by epigenetic DNA methylation (Novakovic et al., 2009; Saffery et al., 2009) and hypermethylation of genes regulating the *VDR* gene may contribute to the reduced placental VDR observed FGR-affected pregnancies. Other potential causes for the decreased VDR in FGR may be due to polymorphism of *VDR*, which are known to affect the expression and function of VDR (Jurutka et al., 2001; Whitfield et al., 2001). VDR polymorphisms have been shown to modify the effect of maternal 25(OH)D on offspring size (Morley et al., 2009).

Several *in vitro* studies have demonstrated that placental Vitamin D and its receptor, VDR play critical roles in the maintenance of normal cellular functions such as proliferation, migration, differentiation and apoptosis. Decreased trophoblast invasion, inadequate remodeling of uterine arterioles (Kaufmann et al., 2003), reduced cytotrophoblast proliferation (Chen et al., 2002), increased cytotrophoblast apoptosis (Crocker et al., 2003), and fusion (Newhouse et al., 2007) are associated with placental insufficiency, which is a key characteristic of FGR pregnancies. Additionally, FGR is characterized by impaired villous trophoblast fusion forming the multi-nucleated syncytiotrophoblast (Kaufmann et al., 2003; Huppertz and Kingdom, 2004; Newhouse et al., 2007). Therefore, decreased placental expression of VDR in the placenta may be a contributing factor to the pathology of idiopathic FGR-affected pregnancies.

BeWo cells, a human choriocarcinoma-derived cell line that is a well-established model for the syncytiotrophoblast, have previously been shown to produce differentiating markers after undergoing syncytialization in the presence of forskolin (an agent increasing cAMP levels) (Mi et al., 2000; Vargas et al., 2008). Pronounced syncytin expression is followed by further cellular differentiation and generation of the syncytium as well as the formation of gap junctions with an associated increase in β-hCG secretion (Frendo et al., 2003; Pathirage et al., 2013). Furthermore, trophoblast syncytialisation is associated with decreased CYP27B1 *in vitro*, (Avila et al., 2004). Previous studies using BeWo cells as a model for syncytiotrophoblast has demonstrated that VDR is a critical regulator of pregnancy and placental hormone secretion including placental lactogen expression and β-hCG (Tuan et al., 1991; Stephanou et al., 1994; Barrera et al., 2008).

In a recent study from our laboratory, we have further confirmed that VDR is involved in BeWo cell differentiation, when treated with a chemical inducer of fusion called forskolin. Differentiation potential of BeWo was evidenced by an increase in β-hCG, which may be due to direct or indirect regulatory role of VDR on β-hCG expression (Nguyen et al., 2015a). In the same study, we have also demonstrated that when VDR mRNA was reduced following siRNA treatment, an increased syncytium formation was observed compared to cells that were treated with non-targeted siRNA control. These observations suggested that VDR inactivation influenced larger syncytium formation *in vitro*. Our study further demonstrated that reduced VDR in these BeWo cells also significantly increased apoptosis, when measured using TP53 mRNA as a marker of apoptosis. Together our own studies suggested the decreased placental VDR may contribute to the increased apoptosis associated with FGR pathogenesis. Alterations in the rate of placental trophoblast and endothelial cell proliferation, differentiation and apoptosis are involved in the pathogenesis of FGR. VDR may confer protection against extrinsic apoptosis and contribute to the maintenance of trophoblast function under adverse conditions, such as chronic hypoxia, inflammation, and under-nutrition, all of which have been linked to FGR. Thus, VDR is likely to be an important regulator of placental functional sufficiency, which is vital for a healthy fetal outcome. However, further studies are warranted to understand the precise cellular and molecular mechanisms by which VDR may contribute to FGR pathogenesis.

## Downstream targets of VDR in the placenta

Feto-placental growth is a complex cascade process requiring tight regulation of cell cycle gene expressions at the transcriptional level. Proliferation, continuous syncytial fusion and a cascade of apoptosis are necessary for the integrity and maintenance of the syncytium layer (Huppertz et al., 1998), and for the remodeling of spiral arterioles by migratory and invasive extravillous cytotrophoblats (EVCTs) (Lyall et al., 1999). Trophoblast proliferation is regulated spatially and temporally throughout pregnancy (Morrish et al., 1998). Proliferation or cell division goes through cell growth and DNA synthesis phases, which are mediated by checkpoints to ensure the cell's fidelity. In our most recent study, (Nguyen et al., 2015b), we identified TGFβ-3, a known inhibitor of cell cycle progression through G1 phase (Caniggia et al., 1999), as a candidate downstream target gene of VDR. Indeed, TGFβ-3 mRNA and protein expression were significantly decreased in placentae from idiopathic FGR-affected pregnancies compared to control placentae. Decreased TGFβ-3 may contribute to inhibition of the G1 phase of a cell cycle resulting in uncontrolled cell proliferation and premature differentiation of cytotrophoblasts, thereby leading to impaired ion and nutrient exchange, and decreased synthesis of hormones required for feto-placental growth and development. Thus, decreased TGFβ-3 caused by changes in VDR signaling may directly or indirectly contribute to placental insufficiency, which is a characteristic of human idiopathic FGR.

**Figure 1:**
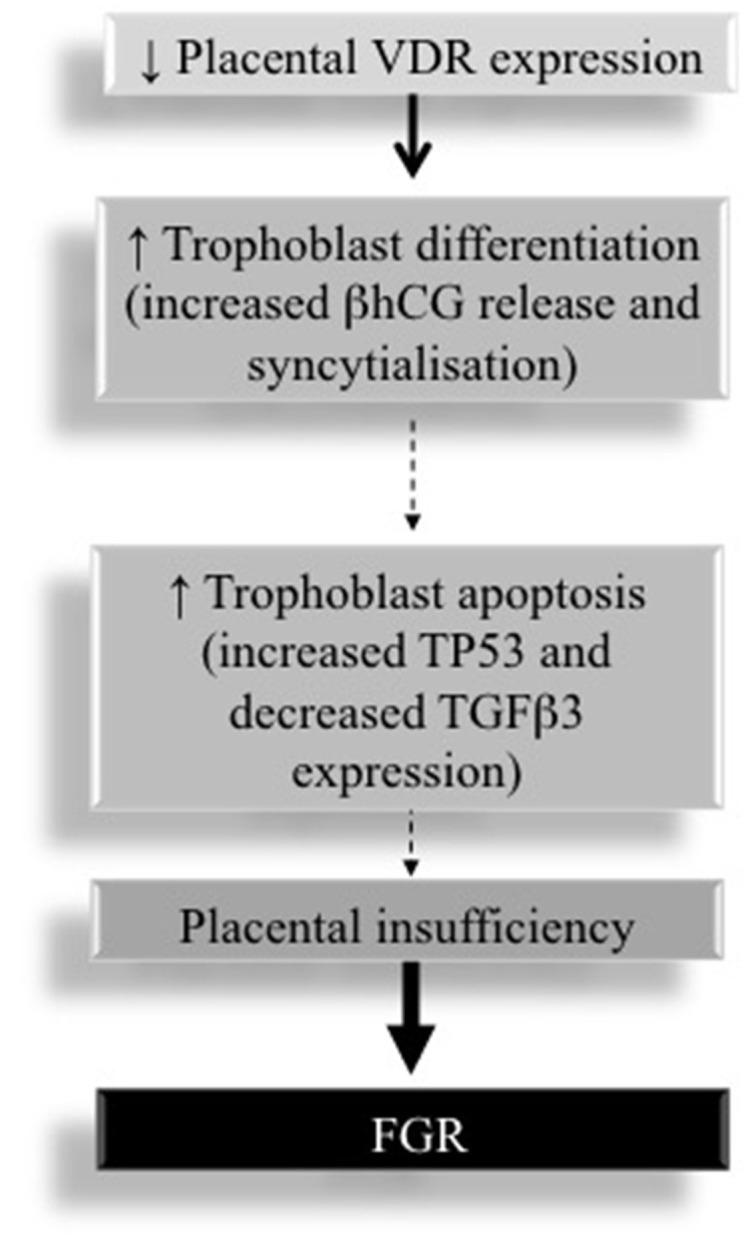
**Placental VDR expression and its contribution to the pathogenesis of FGR**.

As depicted in Figure 1, decreased VDR expression in placentae from FGR pregnancies contribute to alterations in the rate of trophoblast proliferation, differentiation and apoptosis, which are the key features of FGR pathogenesis (Nguyen et al., 2015a). Thus, placental VDR is a critical regulator of feto-placental growth.

## Clinical significance and future directions

If perturbations of placental Vitamin D metabolism, such as decreased placental Vitamin D synthesis and VDR expression, do indeed contribute to FGR, then FGR might be treatable or even prevented through maternal Vitamin D supplementation early in pregnancy. The efficacy of maternally administered Vitamin D supplements should obviously be evaluated more thoroughly, but potentially new intervention strategies that target the placenta might prevent or alleviate altered fetal growth by improving placental Vitamin D metabolism, and thereby improve placental growth and function. Further elucidation of the biological mechanisms by which placental Vitamin D metabolism contribute to FGR will not only increase our understanding of the molecular mechanisms underlying FGR, but may also provide novel approaches to intervention.

## Author contributions

PM conceived the idea, collected all the literature background for the study, designed the experiments, analyzed data and wrote the first draft of the manuscript. HY, TN, SE, HS, RR, MD, HD, DW, EW, and PE assisted with critical data analysis, structure of this review, edited the manuscript.

## Funding

The authors would like to acknowledge the funding support for the project supported by the Australian Institute of Musculoskeletal Science, The University of Melbourne, Australia.

### Conflict of interest statement

The authors declare that the research was conducted in the absence of any commercial or financial relationships that could be construed as a potential conflict of interest.
